# Spin-labeling Insights into How Chemical Fixation Impacts Glycan Organization on Cells

**DOI:** 10.21203/rs.3.rs-3039983/v1

**Published:** 2023-06-13

**Authors:** Mohit Jaiswal, Trang T. Tran, Jiatong Guo, Mingwei Zhou, Sayan Kunda, Zhongwu Guo, Gail Fanucci

**Affiliations:** University of Florida; University of Florida; University of Florida; University of Florida; University of Florida; University of Florida; University of Florida

**Keywords:** Sialic acid, metabolic glycan engineering, chemical fixation, crosslinking, glycan, organization, electron paramagnetic resonance spectroscopy

## Abstract

As new methods to interrogate glycan organization on cells develop, it is important to have a molecular level understanding of how chemical fixation can impact results and interpretations. Site-directed spin labeling technologies are well suited to study how the spin label mobility is impacted by local environmental conditions, such as those imposed by cross-linking effects of paraformaldehyde cell fixation methods. Here, we utilize three different azide-containing sugars for metabolic glycan engineering with HeLa cells to incorporate azido glycans that are modified with a DBCO-based nitroxide moiety via click reaction. Continuous wave X-band electron paramagnetic resonance spectroscopy is employed to characterize how the chronological sequence of chemical fixation and spin labeling impacts the local mobility and accessibility of the nitroxide-labeled glycans in the glycocalyx of HeLa cells. Results demonstrate that chemical fixation with paraformaldehyde can alter local glycan mobility and care should be taken in the analysis of data in any study where chemical fixation and cellular labeling occur.

## 1. Introduction

Cells are shielded by a layer of carbohydrates known as the “cell glycocalyx” that plays an important role in various biological events [1]. Cell surface carbohydrates exist mainly as conjugates with lipids and proteins to assist their attachment onto the cell wall or membrane. Changes in the composition and presentation patterns of glycans in the cell glycocalyx are associated with many diseases [2–4]. For example, more than 80% of cell surface glycoconjugates in the vertebrate brain are glycolipids [5]. It has been revealed that glycosphingolipids (GSLs) are the major component of cell surface glycolipids and are involved in signal transduction [6], cell recognition, adhesion and apoptosis [7, 8], embryonic and nerve system development [9, 10], drug resistance [11], etc. Peculiar GSL expression and presentation are related to cancer [12, 13], diabetes [14], sclerosis [15], Alzheimer’s disease (AD) [16, 17], lysosomal storage disorders [18], and so on. Another type of prominent glycolipids is glycosylphosphatidylinositols (GPIs), whose attachment to proteins is one of the most common posttranslational modifications [19]. GPIs help anchor proteins to the extracellular membrane and are related to many biological activities from signal transduction [20] to cancer [21–24], AD [25], etc. It was also demonstrated that GPI-anchored proteins could not function properly without GPI [26, 27] and that GPI deficiency was lethal [28].

It is generally recognized that the structure of glycans is extremely adaptable to enable their broad functioning via interactions with each other and with other molecules; therefore, the presentation form of glycans on the cell surface can have a decisive impact on their biological activity [29]. As such there is a continued desire to develop novel tools for interrogating glycan organization and mobility on cells. Recently, we demonstrated how nitroxide radical spin labeling with electron paramagnetic resonance (EPR) spectroscopy can be applied to the study of glycans on cells [30–32]. Spin labeling has a rich history of being utilized to probe the local environment in macromolecules and macromolecular assemblies such as lipids, proteins, membrane proteins, nucleic acids, materials like polymers [33–38].

Spin-labeling EPR, which can be performed at physiologically relevant temperatures and conditions, when combined with a spin-labeled biological molecule, provides information on local dynamics, conformations, accessibility, organization, and hydration environment at local sites within biological environments [33, 34, 39–41]. Typically, a persistent radical, such as a nitroxide moiety, is incorporated into the chosen macromolecule (protein, nucleic acids, lipids, polymers etc.) at specific sites of interest. Several different types of spin-labeling magnetic resonance (SLMR) experiments can be performed to provide this information. The mobility of the spin probe (described as a correlation time, $t_{\text{corr}}$, and order parameter, S) [41–43] is determined from lineshape analyses (fitting/simulation) of continuous wave (CW) spectra collected at single or multiple frequencies [44, 45]. Interactions between spin-labeled molecules give rise to changes in lineshapes that arise from dipolar [46] and super-exchange interactions [47, 48], which provide distance information over the range of 6–20 Å from lineshape investigations. Interactions and organization with the membrane and bilayer surface can be measured by power saturation [49–54], and Overhauser dynamic nuclear polarization (ODNP) methods [55–61]. The conformation, dynamics and packing of molecules are reflected quantitatively in these data and can be used to track and quantify changes. As such, SLMR represents an important tool to characterize the molecular environment of glycans in model and cellular membranes that mimic normal or pathological states, as well as to characterize changes that occur upon binding to antibodies or other glycan binding partners.

**Figures 1.**
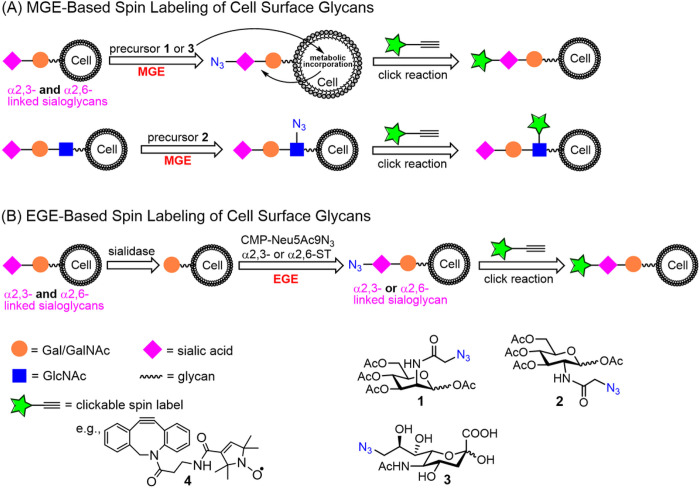
Spin-labelling approach of cell surface sialoglycans via (A) MGE with precursors **1**, **2** or **3** and (B) EGE-mediated incorporation of an azide-modified sialic acid (Neu5Ac9N_3_) residue via either α2,3- or α2,6-siayltransferase (ST) and then a click reaction with DBCO-SL, **4**, to install the nitroxide SL.

Templated site-directed bio-enabled/chemical biological synthesis is often-time utilized for spin labeling in proteins and nucleic acids, whereas fully organic synthetic schemes are more often utilized to incorporate the EPR active radicals into lipids and polymers. Our approach for labeling glycans on cells followed two different chemical biology routes. Specifically, we utilized orthogonal labeling of glycans within the glycocalyx of cells with a click-reactive moiety containing a nitroxide spin label. The first approach involved metabolic glycan engineering (MGE) with an azide-modified modified sugar (Fig. 1A) [30] [62, 63], whereas the second approach exploited enzymatic glycoengineering (EGE) of cell surface glycans with an azide-modified sugar employing different siayltransferases (Fig. 1B) [31, 32].

In these and numerous other cell-based studies, such as immunostaining and genome editing based-strategies or exogenous expression of a tagged protein as well as transmission electron microscopy, a critical methodological step in the sample preparation of some measurements is the fixation of the cell. It is well accepted that chemical fixation of cells disrupts the “live” cell organization/structure [64–67]. Studies are increasing that characterize the molecular level details that chemical fixation has on protein structural organization, such as DNA-protein interactions, liquid-liquid phase separations and emphasis on biomolecules cell and its organelles [68–71], including AFM based investigations of membrane protein clustering during fixation [72]. Here, we investigated the impact that paraformaldehyde (PFA) fixing of cells has on the mobility and organization of glycans in the glycocalyx with EPR spectroscopy. Specifically, we performed spin-labeling studies using MGE approaches of HeLa cells with three different azide-modified monosaccharides, Ac_4_ManAz (**1**), Ac_4_GlcNAz (**2**) and 9-Az-sialic acid (**3**) (Fig. 1), as MGE precursors and varied the order in which cells were spin-labeled and fixed with PFA. EPR spectra were collected and revealed different spectral features that were dependent upon the chronological order of labeling and fixation. Insights into the structural organization giving rise to the different spectral features are inferred from the rates of reduction of the spin label EPR signal by ascorbic acid.

## 2. Materials and Methods

### Materials.

The HeLa cell line was purchased from the American Type Culture Collection (ATCC, United States). The Dulbecco’s modified Eagle’s medium (DMEM), fetal bovine serum (FBS), penicillin-streptomycin (10000 U/mL), and phosphate-buffered saline (PBS) utilized in the study were purchased from Thermo Fisher Scientific (USA). CMP-Neu5Ac9Az, Ac_4_ManAz, and Ac_4_GlcNAz were synthesized using the previously reported method and verified using NMR and MS spectroscopy [73]. Compound **3** (9-Az-sialic acid) was prepared by our lab according to a reported procedure and its ^1^H NMR spectra agreed with that in the literature [63, 74]. DBCO-SL **4** was synthesized following our previously reported protocol [32]. The washing buffer contained 1X DPBS with 2% BSA. Buffer 1 contains 1X DPBS with 20 mM MgCl_2_, 20 mM CaCl_2_, pH7.5.

### Cell culture preparation.

The HeLa cells were maintained in tissue culture flasks under controlled conditions of 37°C, 5% CO_2_, and 95% air, and cultured in Dulbecco’s modified Eagle’s medium (DMEM) supplemented with 100 U per mL of penicillin–streptomycin and 10% fetal bovine serum (FBS). Upon reaching approximately 90% confluency after 3–4 days of culture, the cells were harvested from the culture surface by Gibco^™^ Trypsin-EDTA (0.25%) and subsequently suspended in a fresh culture medium. An aliquot of cell suspension was used to count cell numbers using a 0.4% trypan blue stain and a hemocytometer. Following that, the required number of cells were transferred to a new culture flask and subjected to the metabolic engineering experiment or directly transferred to a centrifuge tube for chemoenzymatic engineering experiment.

### Metabolic engineering-based spin labeling of Hela cells.

Ac_4_ManAz (**1**) or Ac_4_GlcNAz (**2**) stock solution (40 μL, 20 mM stock in ethanol) was transferred to a T-25 tissue culture flask (25 cm^2^), and the flask was placed in a sterile laminar hood to allow the ethanol to evaporate. Then, 4 mL of DMEM media supplemented with 100 U/mL of penicillin–streptomycin and 10% FBS was added to each flask to reach the desired concentration of 200 μM of **1** or **2**. For engineering using 9-Az-sialic acid (**3**), the required amount (8 μL, 100 mM) of stock in DPBS was directly added to the cell culture media. For negative control, cells were incubated in culture media without **1**, **2**, or **3**. After adding 1.5 million cells to each flask, the cells were cultured for 48 hours under the previously described conditions. The cells were then harvested by adding 750 μL of an EDTA solution (1 mM stock in dd water) in PBS (pH 7.4) to each flask, followed by 8 minutes of centrifugation at 300 rpm and 4°C to acquire the cell pellet. After that, the cells were washed with washing buffer three times and counted as described above. Finally, the cells were resuspended in a 4% paraformaldehyde (PFA) solution (in PBS, pH 7.5) for 20 min at RT, washed 3 times with washing buffer, and subjected to spin label installation (For cells designated as Fix > SL).

For cells designated as SL > Fix, the DBCO-SL reaction was performed before incubation with 4% PFA, with all the other steps identical. The cell pellet was resuspended in 200 μL of washing buffer containing 100 μM DBCO-SL (**4**) (20 μL of 1 mM stock in DMSO). The cell suspension was then incubated in a shaker at RT for 1 h with shaking at 300 rpm with occasional pipetting of the air to prevent cells from settling. The reaction was quenched by adding excess ice-chilled washing buffer, followed by centrifugation, and washing three times. Finally, the cells were pelleted down by centrifugation at 6000 rpm (2300 × g) for 1 min, and the cell pellet was subjected to EPR analysis.

### MGE-based fluorescent labeling of cells and flow cytometry analysis.

The Hela cells experienced metabolic engineering through the application of 200 μM of a metabolic reporter molecule **2**, following the exact same procedures as described above. Cells were harvested and washed three times with ice-cold washing buffer, then resuspended in 100 μL of washing buffer containing 50 μM of DBCO-FAM (Fluorescein dibenzocyclooctyne, Lumiprobe Corp. Hunt Valley, MD) and kept at RT in the dark for 60 mins with occasional air agitation to the cells. Thereafter following a quick washing with the washing buffer, the cells were incubated in 100 μL of 4% paraformaldehyde solution (in PBS, pH 7.5) for 20 mins at RT. Finally, the cells (designated as Click + Fix) were washed again three times with the washing buffer before being analyzed on the flow cytometer. An Attune^™^ Nxt flow cytometer was used with the blue excitation (BL1) laser (Ex: 488 nm, Em: 520 nm), and the data was analyzed using Attune^™^ Nxt software. For the cells designated as Fix + Click, the fixing step with 4% PFA was performed prior to the click conjugation process, with all other steps remaining identical. Control samples were subjected to labeling with DBCO-FAM without treatment of 2.

### Ascorbic acid quenching of spin labels.

Ascorbic acid quenching of the spin probe was performed following a previously reported method [75]. Using the above-described method, HeLa cells were engineered, then fixed and spin-labeled (or spin-labeled and then fixed). In both the cases, cell pellets were resuspended in 1X DPBS containing 2.30 mM L-ascorbate (9.2 μL from a 50 mM stock in 1X DPBS) in a final volume of 200 μL and then incubated at room temperature (RT) for various time intervals with occasional air agitation in the tube. Samples from different time points were washed three times with an excess ice-cooled washing buffer, and the cell pellet was subjected to EPR analysis.

### CW-EPR data collection and analysis of spin-labeled cells.

X-band (9.5 GHz) CW-EPR absorption spectra were collected at 30°C using a Magnettech MiniScope MS-5000 bench-top EPR spectrometer with a dielectric resonator. Spectra were reported as an average of 16 scans with 120 mT sweep width, 0.2 mT modulation amplitude, 100 kHz modulation frequency and 1 mW incident microwave power (2 mW incident microwave power on Bruker E500). All of the EPR spectra were area normalized to the cell number, and all spectra were baseline-corrected and processed using the LabVIEW software provided by C. Altenbach and W. Hubbell (https://sites.google.com/site/altenbach/labview-programs).

### Lineshape analysis and simulation of EPR spectra.

EPR spectra were simulated using the chili and esfit functions of EasySpin [76] as done in our previous studies using DBCO-SL [30–32]. The A- and g-tensors were previously determined: $g_{xx}=2.0070,\;{\;g}_{yy}=2.0062,\;{\;g}_{zz}=2.0033,\;{\;A}_{xx}=6.7\;G,\;A_{yy}=6.7\;G$, and $A_{zz}=35\;G$ [30]. The other parameters used in EPR lineshape simulations are linewidth, correlation time of motion $\left(t_{c}\right)$, and the ordering potential C20. Each EPR spectrum was subjected to 1-, 2-, and 3-component simulations.

## 3. Results and Discussion

**Figure 2.**
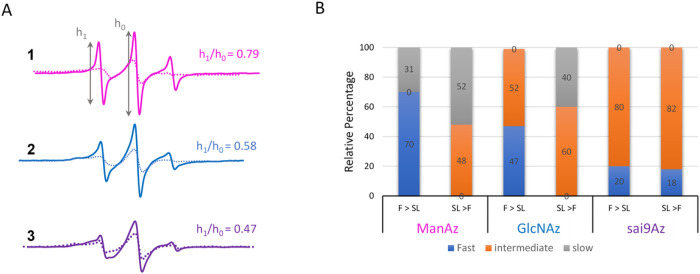
Stack plot of vertically offset 100G X-band EPR spectra for control-subtracted HeLa cells treated with Ac_4_ManAz (**1**), Ac_4_GlcNAz (**2**) or 9-Az-Sialic acid (**3**) that were fixed with PFA and then reacted with DBCO-SL **4** (F>SL, solid lines) or reacted with DBCO-SL **4** and then fixed with PFA (SL>F, dotted lines). Spectra are plotted with normalized integral areas to reflect mobilities. Annotations for the $h_{1}$ and $h_{0}$ transition area shown. Values for $h_{1}/h_{0}$ given in the figure are evaluated for spectra from conditions where the cells were fixed and then spin-labeled (F>SL). (B) Relative results of spectral components from fitting with Easy Spin where $t_{R}\leq 1.5\;ns$ is designated as “fast”, $1.5\;ns<t_{R}\leq 8\;ns$ is designated as “intermediate”, and $t_{R}>8\;ns$ is designated as “slow”. Numbers reflect the relative percentages.

MGE with Ac_4_ManAz (**1**), Ac_4_GlcNAz (**2**) or 9-Az-Sialic acid (**3**) was performed with analogous control samples that did not contain the azide-modified sugars but were subjected to the same procedure of fixing/spin labeling with DBCO-SL (**4**). Control samples resulted in a background EPR signal of non-specifically interacting labels [30]. As done previously, these cell-count normalized control spectra are subtracted from the cell count normalized labeled spectra. Figure 2 shows the resultant control subtracted EPR spectra for MGE HeLa cells prepared with varied chronological order of labeling and chemical fixation with PFA. Spectra of HeLa cells treated with precursors **1** and **2** that were fixed before labeling are consistent with earlier published data [30]. EasySpin was used to fit the experimental results [76], and spectra are well fit by two components where one spectrum reflects a more mobile lineshape and the other a more immobilized local environment for the spin label (Fig. 2 and Fig S1, Table S1). For purposes of comparison within, we have designated three different motional regimes with $t_{R}\leq 1.5\;ns$ as “fast”, $1.5\;ns<t_{R}\leq 8\;ns$ as “intermediate”, and $t_{R}>8\;ns$ as “slow”, where the relative fractions of these components vary depending upon the sugar identity and order of PFA fixation and spin labeling.

Here we report MGE results for the first-time using precursor **3**. EPR results for **3** indicate an on average more restricted environment with a complex lineshape of multiple components and only a small fraction of the mobile $t_{R}\sim 1$ ns component. At first, this result is somewhat surprising given that both **1** and **3** are expected to be incorporated into glycan biosynthesis as sialic acids (Fig. 1), and as such we anticipated similar EPR spectra for cells treated with **1** and **3**. However, the location of the azide differs in **1** and **3**. Furthermore, **1** and **3** experience different metabolic processes, thus there are significant differences between ManNAc with regard to sialic acids regarding their metabolism, incorporation efficiency, selectivity for sialoglycans and chemical modification potential [77]. All these factors may affect the final expression level and pattern of azido sialoglycans on the cell surface, thus the EPR results. Additionally, we recently showed that incorporation of Neu5Ac9N_3_ with either an α2,3- or α2,6-siayltransferase (ST) resulted in different resultant EPR lineshapes based upon organizational differences that could limit mobility of the rotatable bonds of the spin label, the mobility of the azide-modified sugar, and/or glycosidic bond mobility. EPR lineshapes for MGE with **3** differ from that using **1** or **2** in that they possess a relatively small percentage of a highly mobile site with a more restricted site characterized here as having slow mobility. Irrespective of the complexity, the EPR spectrum of spin-labeled cells treated with **3** reveals the lowest mobility $\left(h_{1}/h_{0}=0.47\right)$.

For all metabolic precursors used, the lineshape for the EPR spectra that were spin labeled before treatment with PFA reflect lower mobility than when spin-labeled after chemical fixation. Spectra in Fig. 2 are plotted with signal intensity normalized to area, where broadened spectra have less intensity and reflect overall lowered mobility [33, 34, 41, 78]. More insights into the local mobility of the spin-labeled glycans is afforded from theoretical spectral fitting [76]. For example, with **2**, the spectrum resulting from fixing prior to spin labeling contains about 47% of the “fast” mobile component with the remaining signal comprising an intermediate motion component. In contrast, when cells treated with **2** are spin-labeled and then fixed, the EPR results show about 60% of an intermediate component and 40% of a slow component with no spectral components reflective of fast isotropic motion (Fig. 2B). Perhaps these results are not surprising. The chemical fixation process with PFA cross-links together proteins on the cell surface and if done prior to spin labeling may afford more sites clearly exposed on the outer sphere of the cell glycocalyx for spin labeling. In contrast, chemical fixation after spin labeling could cluster together the already spin-labeled glycoproteins and entrap labeled glycolipids, giving rise to a higher percentage of restricted glycans. More important, very similar spectra irrespective of the identity of the azide-modified sugar used in the MGE are obtained when chemical fixation occurs after spin labeling. Thus, differences in the glycan organization, reflected in the EPR spectral lineshapes, are only revealed for **1** and **2** when spin labeling occurs after the chemical fixation reactions.

**Figure 3.**
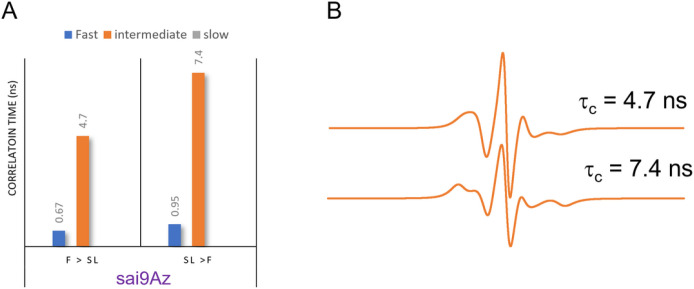
(A) Correlation time analysis from theoretical fitting of MGE of HeLa cells treated with **3**as a function of the order of chemical fixation and (B) intermediate motion EPR component spectra from Easy Spin fitting showing how the EPR lineshape has discernable changes with respect to the intermediate regime correlation times.

EPR of spin-labeled cells using **3** for MGE shows slightly different results than obtained for **1** and **2**. A small fraction of a fast mobility component is present in the spectrum from treatment with **3** even when SL is performed prior to PFA fixation. The total percentage of the fast mobility components for **3** is ~ 20%. When comparing results obtained when chemical fixation occurs before spin labeling, the relative fraction of the fast mobility component is less than those observed with either **1** (73%) or **2** (47%). Although the presence and relative percentages of fast mobility and intermediate mobility component with MGE treatment of **3** do not differ with the order of chemical fixation, the mobility of the spectral components within a given motional regime differs. The results from simulation of cells treated with **3** with varying order of chemical fixation and labeling are shown in Fig. 3. The mobility of both components decreases upon fixation after spin labeling (*i.e*., a larger correlation time means slower motion). As inferred from CW-/X-band EPR lineshape differences, the order of chemical fixation manifests less of an effect on glycan organization and mobility when using **3** compared to **2** or **1** for MGE with HeLa cells.

**Figure 4.**
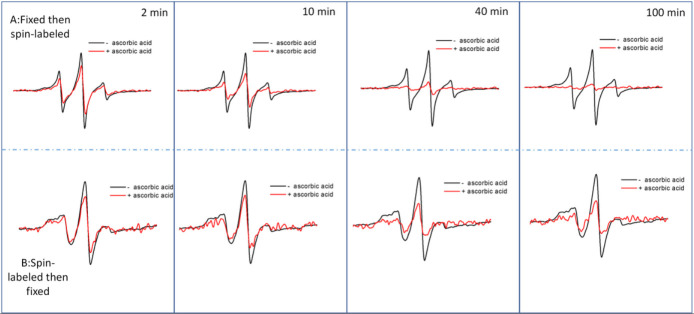
X-band EPR spectra for control subtracted HeLa cells treated with Ac_4_GlcNAz (**2**) and DBCO-SL before (black) and after (red) exposure to ascorbic acid over the time course of 100 minutes. Spectra after exposure to ascorbic acid represent only 2 scans of signal averaging, whereas the before spectra have better signal-to-noise ratios because they were signal averaged for 16 scans. Spectra are plotted normalized to total cell count, so the decrease in signal is reflective of the decrease in EPR active radical that remains in the sample.

**Figure 5.**
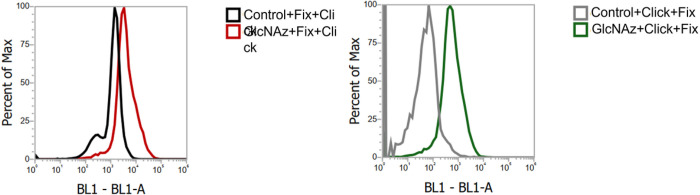
FLACS results demonstrating increase in fluorescence intensity of HeLa cells treated with **2**and labeled with DBCO-FAM which demonstrate that greater fluorescence is observed when labeled prior to fixing than vice-versa.

To further probe organizational differences that arise from the order of fixation relative to spin labeling, we subjected samples to reduction with ascorbic acid. Ascorbic acid will reduce the nitroxide radical, thus diminishing the intensity of the EPR signal [75]. The ability of ascorbic acid to penetrate the cross-linking extracellular matrix is dependent upon the accessibility and diffusion ability of the small molecule through the crosslinked glycocalyx. This reduction process occurs on the minute time scale, so we can take aliquot samples of cells from a batch reaction, quench with cold water and collect EPR scans. Figure 4 shows a set of spectra obtained for HeLa cells treated with Ac_4_GlcNAz (**2**) with different orders of fixing and spin labeling over a 100-minute time span. We note that the total EPR signal from equivalent number of cells that were spin-labeled and then fixed was approximately only ~ 40% total intensity compared to cells that were fixed and then spin-labeled. We attribute this reduction in total EPR signal to chemical reaction of the nitroxide with the chemical reagents during PFA cross linking. In contrast, we performed analogous studies with DCBO-fluorophores, which demonstrate increased fluorescence intensity when labeled prior to cross linking – an opposite trend (Fig. 5). As such, we interpret these results to suggest that more of the azide-modified glycans are available for click-reaction when labeling is prior to fixation, but the chemistry of the fixation process can chemically reduce a marked fraction of the total nitroxide radicals incorporated into the glycocalyx.

**Figure 6.**
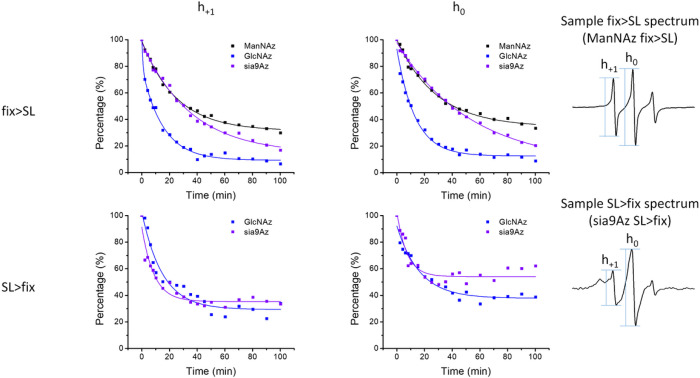
Resultant time course data of ascorbic acid reduction of spin-labeled HeLa cells with Ac_4_ManAz (**1**), Ac_4_GlcNAz (**2**) or 9-Az-Sialic acid (**3**) and DBCO-SL and evaluated by both the intensities of the $h_{1}$ and $h_{0}$ transitions. Values of $h_{1}$ and $h_{0}$ at t=0 were acquired prior to addition of ascorbic acid and were set to 100% after scaling for dilution required by adding ascorbic acid to cells for additional time step measurements. All other intensities were scaled accordingly.

Because of the lowered signal-to-noise ratios in the kinetic studies, we did not attempt to simulate the spectra acquired as a function of time in the presence of ascorbic acid. Nevertheless, the spectral lineshape does not appear to change over the time course investigated (Fig S2). Only the total intensity is observed to markedly change over time. Because of this, the kinetic process of ascorbic acid reduction was evaluated by tracking the percentage remaining intensity of the $h_{1}$ and $h_{0}$ spectral features, relative to prior to ascorbic acid addition, as a function of time. Figure 6 plots results for ascorbic acid reduction for labeled HeLa cells treated with **1**, **2** or **3** with varying order of fixing/spin labeling, where solid lines are fits to the data using a decay function with either one or two independent exponential decay rates,

$$y=A_{1}e^{\left(-\frac{x}{t_{1}}\right)}+y_{0}$$

$$\;y=A_{1}e^{\left(-\frac{x}{t_{1}}\right)}+A_{2}e^{\left(-\frac{x}{t_{2}}\right)}+y_{0}$$

Where $A_{1}$ and $A_{2}$ reflect the relative percentages of each decay component, $y_{0}$ represents the relative percentage of remaining intensity after 100 minutes of exposure to ascorbic acid and $t_{1}$ and $t_{2}$ are the respective decay time constants.

**Figure 7.**
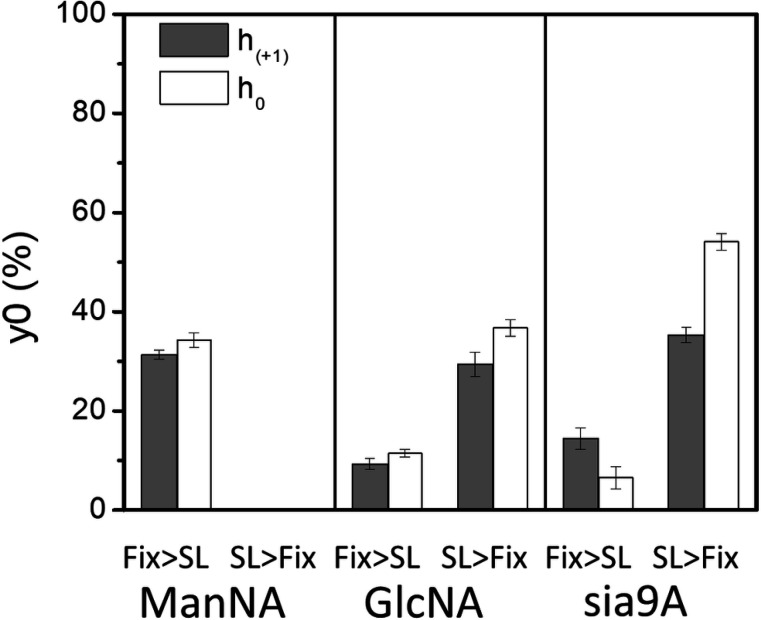
Resultant relative percentage of remaining EPR active spins after 100 min exposure to ascorbic acid for HeLa cells treated with Ac_4_ManAz (**1**), Ac_4_GlcNAz (**2**) or 9-Az-Sialic acid (**3**) and DBCO-SL, where the order of fixing and spin-labeling was altered.

Table 1 summarizes the results from fitting of the decay curves. In most cases a single component exponential fit was only necessary for suitable fitting of the data. In terms of accessibility, results indicate that for both **2** and **3**, approximately 90% of the total signal was reduced (*i.e*.,10% remaining) after 100 minutes of exposure to ascorbic acid when cells were chemically fixed prior to spin labeling, whereas only 60–70% of the signal was reduced (*i.e*.,30–40% remaining) when spin-labeled prior to chemical fixation, thus, indicating that the crosslinks among biomolecules on cells that lead to broadened EPR spectra also make the SL less accessible to ascorbic acid under these same conditions (Fig. 7). The results are quite similar for both the $h_{+1}$ and $h_{0}$ signals, again supporting that the relative populations of the mobile and immobile components (for spectra Fix prior to SL) do not change significantly throughout the time course of these experiments or that both features have roughly equal accessibility to ascorbic acid. The reasoning follows that the central line has the greatest contributions from both components and the $h_{+1}$ and $h_{-1}$ will be more dominated by the relative fraction of the slower component.

**Figure 8.**
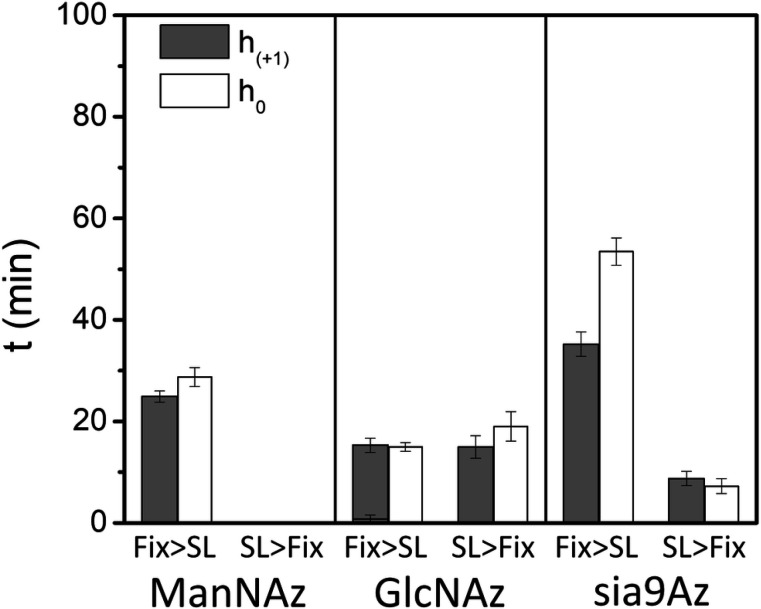
Resultant kinetic time constants for ascorbic acid reduction of the EPR spectrum from HeLa cells treated with Ac_4_ManAz (**1**), Ac_4_GlcNAz (**2**) or 9-Az-Sialic acid (**3**) and DBCO-SL, where the order of fixing and spin labeling was altered.

In terms of the decay times, the order of reduction in EPR signal followed the trend of **2** was the fastest, followed by **1** and then **3** when cells were chemically fixed and then spin-labeled, where in contrast when the cells were spin-labeled and then chemically fixed (Fig. 8), **3** now had the fasted reduction time constant. These findings suggest disparate impacts on the organization and accessibility of various types of glycans in the glycocalyx when labeled either prior to or after cell fixation.

Because of this variability, we had since chosen to pursue enzymatic labeling of cells with orthogonal click reactive chemistry where no chemical fixation of cells was utilized in the protocol. However, we were unable to obtain similar high quality reproducible EPR signals from MGE cells that were spin-labeled and not chemically fixed. Through engineering of EPR sample holders that can maintain 4 °C, it may be possible to inhibit endocytosis and reduction of the spin label; yet we want to develop SDSL methods that can be utilized at physiological temperatures. In this case, a less perturbing cell fixation method is desirable. A recent review of the impacts of chemical fixation on the cellular contents of cells has proposed future directions for the development of potentially less perturbing chemical fixatives [68].

## 4. Conclusions

Taken together, the spin labeling EPR approach is exquisitely well-suited to show how cell fixation impacts the resultant EPR lineshape, which reflects changes in the average mobility and organization of the spin-labeled glycans on the surface of cells. The accessibility measurements also indicate differences in the local environment of the spin labels depending upon the order of treatment with crosslinking agents. These results have clearly demonstrated that the cell fixation protocol can have a significant impact on the organization and mobility of components within the cellular glycocalyx, the accessibility of cell surface glycans for molecular labeling, and the properties and dynamics of labeled glycans. As such, it is suggested that care should be taken when analyzing results of membrane organization obtained from any method where chemical fixation of cells is performed.

## Figures and Tables

**Table 1:** Decay rate parameters for ascorbic acid reduction of the nitroxide EPR signal.

Sugar:	ManNAz (1)				GlcNAz (2)				Sia9Az (3)			
Order:	Fix > SL		SL > Fix		Fix > SL		SL > Fix		Fix > SL		SL > Fix	
transition	$h_{+1}$	$h_{0}$	$h_{+1}$	${\;h}_{0}$	$h_{+1}$	${\;h}_{0}$	$h_{+1}$	$h_{0}$	$h_{+1}$	${\;h}_{0}$	$h_{+1}$	${\;h}_{0}$
$y_{o}$	31.3 ± 0.9	34.3 ± 1.4	--^a^	--^a^	9.2 ± 1.1	11.5 ± 0.8	29 ± 2	37 ± 2	15 ± 2	7 ± 2	35 ± 2	54 ± 2
$A_{1}$	67.8 ± 1.0	65.5 ± 1.5	--^a^	--^a^	69 ± 3	77 ± 2	73 ± 4	52 ± 3	84 ± 2	91 ± 2	56 ± 4	49 ± 5
$t_{1}$	25 ± 1	29 ± 2	--^a^	--^a^	15 ± 2	15 ± 1	15 ± 2	19 ± 2	35 ± 2	53 ± 2	9 ± 2	7 ± 2
$A_{2}$	*n.a.*	*n.a.*	--^a^	--^a^	22 ± 4	12 ± 3	*n.a.*	8 ± 5	*n.a.*	*n.a.*	*n.a.*	*n.a.*
$t_{2}$	*n.a.*	*n.a.*	--^a^	--^a^	*0.07* ^c^	*0.02* ^c^	*n.a.*	*0.08* ^c^	*n.a.*	*n.a.*	*n.a.*	*n.a.*

a These data were not collected.

b A second exponential was not needed to adequately fit the data.

c These decay components were too fast for accurate measurements given our first time point was longer than this decay time. Nevertheless, this second component was required for adequate fitting of the data.
